# Survival outcomes and contributing factors in oral squamous cell carcinoma patients in Khuzestan province, southwest of Iran

**DOI:** 10.3389/fonc.2024.1472190

**Published:** 2024-12-16

**Authors:** Babak Karimi, Arian Ghoreyshvandi, Maria Cheraghi

**Affiliations:** ^1^ Department of Oral and Maxillofacial Pathology, School of Dentistry, Ahvaz Jundishapur University of Medical Sciences, Ahvaz, Khuzestan, Iran; ^2^ Social Determinants of Health Research Center, Department of Public Health, School of Health, Ahvaz Jundishapur University of Medical Sciences, Ahvaz, Khuzestan, Iran

**Keywords:** oral, squamous cell carcinoma, OSCC, survival, cancer, Iran

## Abstract

**Background:**

Oral squamous cell carcinoma (OSCC) is the most frequent oral cancer worldwide. Despite advances in OSCC treatment, the mortality rate has not decreased in recent years. Therefore, the aim of this investigation was to assess the survival rate as a factor reflecting the quality aspects of care and background parameters that influence survival in patients with OSCC.

**Methods:**

This is a retrospective analysis of 165 patients with OSCC who were registered in the Khuzestan cancer registry system in 2014 to 2018. The data were collected in two parts: demographic information and survival information. Demographic and background variables include age, gender, marital status, ethnicity, employment status, insurance status, and educational status. Survival information was also collected through phone calls to patients or their families. The survival rate of the patients was analyzed using the log-rank test and the influencing factors were analyzed with the Cox regression test.

**Results:**

In this study, 165 patients, 43 women (26.1%) and 122 men (73.9%), with OSCC were included. The follow-up period of the patients was 5 years (2014–2019), during which 74 patients died. One, three, and five-year survival rates were 93.34%, 71.51%, and 44.84%, respectively. The results showed that age (χ^2^ = 4.410, *p* < 0.05) and employee status (χ^2^ = 10.205, *p* < 0.05) were associated with survival rate in OSCC patients based on the log-rank test results, while Cox regression analysis, after including all variables in the model and adjusting them, showed that all variables were not associated with survival rate (*p* > 0.05).

**Conclusion:**

Since all background factors were not associated with survival rate, efforts should continue to identify effective factors and investigate the relationship between survival and pathological factors such as recurrence status, distant metastasis, type of treatment, and involved organs.

## Introduction

Oral cancer comprises a group of neoplasms that involve any area of the oral cavity, oropharynx, and salivary glands (1). However, this term is often used instead of oral squamous cell carcinoma (OSCC), which represents the most common neoplasms of the mouth. It is estimated that over 90% of all oral neoplasms are of the OSCC type (2). In the United States, more than 30,000 patients are diagnosed annually with various types of oral cancers, with over 8,000 individuals dying due to cancer (3).

The data from the Global Cancer Observatory indicate that the annual incidence of OSCC in 2020 was 377,713 cases worldwide, with the highest number in Asia (248,360), followed by Europe (65,279) and North America (27,469). The 5-year prevalence of OSCC is estimated to be close to 1 million (959,248) and follows the same pattern of incidence (4). The available data on the trend of OSCC in Iran is extremely limited and inadequate. Furthermore, there has been a lack of updates on the epidemiological data of OSCC in recent years.

However, according to the scientific report published by the Islamic Republic of Iran Ministry of Health in 2003, oral cancer ranks among the top 10 prevalent cancers in three provinces for women and in five provinces for men, out of all the cancers that have affected both genders (5).

Multiple clinical, pathological, and molecular markers play a role in predicting the prognosis of oral cancer. Various studies have been conducted to determine prognostic factors in patients with OSCC and showed that factors such as location, stage, time from diagnosis to treatment, local recurrence, tumor differentiation, degree of keratinization, and pattern of lymph-vascular invasion have a significant correlation with 5-year survival (6, 7). In a retrospective study, Narges Gholizadeh et al. examined predictive factors for survival rate in OSCC in Iran. Data collection was from medical records of patients with oral cancer from 2009 to 2012 in the cancer department of the Islamic Republic of Iran Ministry of Health. The finding showed that the overall 5-year survival rate was 40.24% (SE = 5.5). Moreover, age and regular follow-up had a statistically significant relationship with survival rate (8).

OSCC is usually diagnosed in advanced stages; thus, despite advances in diagnostic and treatment methods, the mortality rate remains high (9). Public health professionals have long been focusing on the survival rate as an indicator of quality of screening, diagnostic, and treatment programs. Hence, according to the limited number of survival studies in southern Iran, the current study was conducted using cancer registry data in Khuzestan province from 2014 to 2018 to estimate the overall 5-year survival rate of OSCC in the Khuzestan province and identify associated demographic and background factors to understand the current situation, enable national and international comparisons, and plan strategies to control this disease in the future.

## Material and methods

### Patient and data collection

The current study was a cross-sectional study conducted using a descriptive–analytical method. The study population consisted of all patients with OSCC who were registered in the Khuzestan cancer registry system from 2014 to 2018. The sampling method was census. The data collection was divided into two parts. The first part included background parameters of the patients, such as age (under 55, greater than or equal to 55), gender (male, female), marital status (single, married), ethnicity, education (illiterate, below diploma, diploma and above), employment status (housewife, retired, employee, others) and insurance type (public health, supplemental health), which were collected by referring to the Khuzestan cancer registry database. The second part—information about the overall 5-year survival rate—was collected through phone call after obtaining the consent of the patients or their relatives and ensuring confidentiality. The inclusion criteria for the study included individuals’ willingness to participate in the study. Individuals who did not complete the questionnaire and those who had a history of systemic chronic diseases at the time of cancer diagnosis were excluded from the study. This study was approved by the Ethics Committee in Ahvaz Jundishapur University of Medical Sciences (ethics code: IR.AJUMS.REC.1402.201).

### Statistical analysis

The data were analyzed using the statistical software SPSS 26 (IBM SPSS Statistics, Armonk, NY, USA). Data were presented as frequency (percentage) for categorical variables. The follow-up period began after the initial treatment for each patient. It ended when the patient expired, experienced tumor recurrence, or was lost to follow-up. The significance of the curves was assessed using the log-rank test. Potential risk factors and pathological characteristics were further analyzed using a multivariate Cox regression model to obtain independent predictors of survival. The final model was constructed in stages, and differences in clinical characteristics were examined using the χ^2^ test. In all analyses, *p* < 0.05 was considered significant.

## Results

### Patient characteristics

In this study, 165 patients, 43 women (26.1%) and 122 men (73.9%), with OSCC who were undergoing chemotherapy treatment were included. Patients were divided into five ethnic groups: Lur (11.5%), Fars (15.8%), Arab (53.9%), Turk (5.5%), and Bakhtiari (13.3%). Patients were categorized into two groups: over 55 years old (35.2%) and under 55 years old (64.8%). Additionally, individuals were divided into three educational categories: illiterate (13.3%), below diploma (42.4%), and diploma and above (44.2%) (**Table 1**).

**Table 1:** Characteristics of 165 patients with oral squamous cell carcinoma.

Variable	Frequency (%)
Age	
>55	107 (64.8)
≤55	58 (35.2)
Gender	
Male	122 (73.9)
Female	143 (26.1)
Marital status	
Single	142 (86)
Married	23 (13.9)
Education status	
Illiterate	22 (13.3)
Below diploma	70 (42.40)
Diploma and above	73 (44.2)
Ethnicity	
Lur	19 (11.5)
Arab	89 (53.9)
Bakhtiari	22 (13.3)
Turk	9 (5.5)
Fars	15.8 (26)
Employment status	
Housewife	27 (16.4)
Retired	50 (3.3)
Employee	58 (35.2)
Others	3. (18.2)
Insurance status	
Public health insurance	93 (56.4)
Supplemental health insurance	50 (30.3)
Without insurance	13.3 (13.3)

### Survival analysis

**Figure 1:**
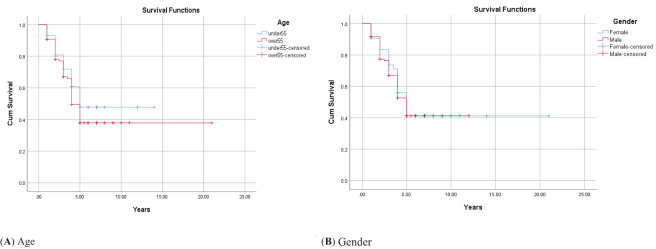
Adjusted parametric survival curves illustrating OSCC-specific survival by age **(A)** and gender **(B)**, *p* < 0.001 by the log-rank test.

**Figure 2:**
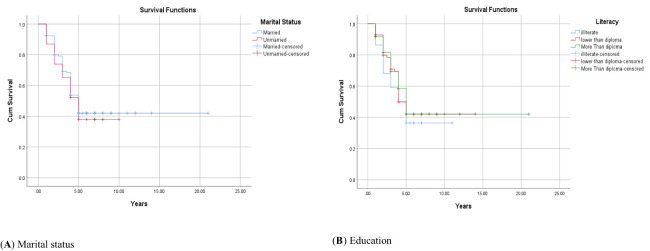
Adjusted parametric survival curves illustrating OSCC-specific survival by marital status **(A)** and education **(B)**, *p* < 0.001 by log-rank test.

**Figure 3:**
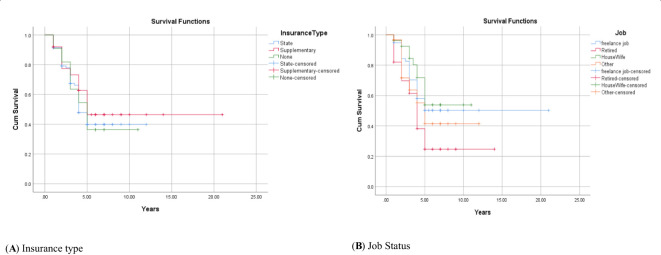
Adjusted parametric survival curves illustrating OSCC-specific survival by insurance type **(A)** and job status **(B)**, *p* < 0.001 by log-rank test.

**Figure 4:**
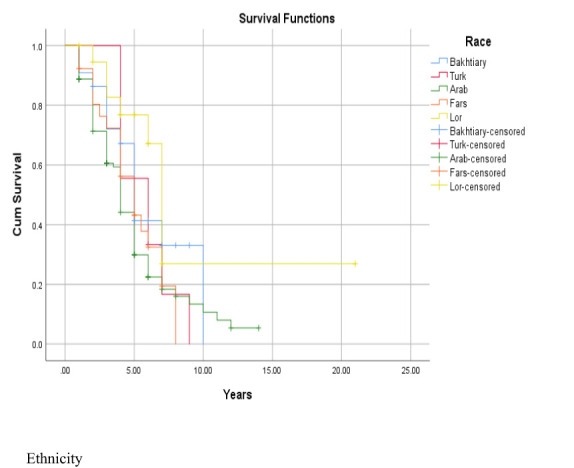
Adjusted parametric survival curves illustrating OSCC-specific survival by ethnicity, *p* < 0.001 by log-rank test.

The 1-, 2-, 3-, 4-, and 5-year survival for OSCC was 93.34%, 81.21%, 71.51%, 56.96%, and 44.84%, respectively. Based on the log-rank test results in **Table 2**, age (df = 2, χ^2^ = 4.410, *p* = 0.036) and employment status (df = 2, χ^2^ = 10.205, *p* = 0.017) were found to be associated with the overall 5-year survival rate, while other variables such as gender, marital status, ethnicity, insurance status, and education status were not associated with the overall 5-year survival rate (**Figures 1**–**4**).

**Table 2:** The participants’ survival experience based on demographic variables.

Variable	χ2*	Degree of freedom (df)	*p*-value**
**Age**	4.41	1	0.036**
**Gender**	2.533	1	0.111
**Marital status**	0.017	1	0.897
**Education status**	1.863	2	0.394
**Ethnicity**	8.551	4	0.073
**Employment status**	10.205	3	0.017**
**Insurance status**	1.913	2	0.384

*The log-rank (Mantel-Haenszel), **p-value < 0.05.

The results of Cox regression analysis, after including all variables in the model and adjusting them, indicate that none of them were statistically significant (*p*-value > 0.05). Therefore, we sequentially removed each variable from our model, starting with the ones with higher *p*-values, and obtained the results shown in **Table 3**. However, all variables were not to be significant. This analysis was using the “backward method”, which consists of six steps, as displayed in **Table 3**. Based on the findings presented in **Table 3**, it is evident that the hazard ratio (HR) for various variables was close to 1, especially during the initial stages of Cox regression.

**Table 3:** Cox regression using the “backward method” shows hazard ratios of variables in each step.

		B	SE	Wald	df	*p*-value	HR	95.0% CI for HR	
								Lower	Upper
Step 1	Gender	−0.518	0.321	2.606	1	0.106	0.596	0.317	1.117
	Age	−0.535	0.328	2.657	1	0.103	0.586	0.308	1.114
	Job	0.008	0.134	0.004	1	0.950	1.008	0.775	1.311
	Literacy	0.389	0.266	2.146	1	0.143	1.476	0.877	2.483
	Race	0.143	0.132	1.187	1	0.276	1.154	0.892	1.494
	Insurance Type	−0.137	0.237	0.333	1	0.564	0.872	0.548	1.389
	Marital status	−0.396	0.472	0.701	1	0.402	0.673	0.267	1.699
Step 2	Gender	−0.523	0.313	2.783	1	0.095	0.593	0.321	1.096
	Age	−0.536	0.327	2.682	1	0.101	0.585	0.308	1.111
	Literacy	0.392	0.261	2.266	1	0.132	1.480	0.888	2.467
	Race	0.144	0.131	1.199	1	0.274	1.155	0.893	1.494
	Insurance Type	−0.136	0.237	0.329	1	0.566	0.873	0.549	1.388
	Marital status	−0.403	0.458	0.774	1	0.379	0.668	0.272	1.640
Step 3	Gender	−0.491	0.309	2.525	1	0.112	0.612	0.334	1.121
	Age	−0.525	0.326	2.594	1	0.107	0.591	0.312	1.121
	Literacy	0.417	0.257	2.636	1	0.104	1.518	0.917	2.512
	Race	0.145	0.131	1.231	1	0.267	1.156	0.895	1.495
	Marital status	−0.393	0.456	0.746	1	0.388	0.675	0.276	1.648
Step 4	Gender	−0.494	0.310	2.551	1	0.110	0.610	0.332	1.119
	Age	−0.449	0.317	2.013	1	0.156	0.638	0.343	1.187
	Literacy	0.402	0.255	2.494	1	0.114	1.495	0.908	2.463
	Race	0.143	0.131	1.191	1	0.275	1.153	0.893	1.491
Step 5	Gender	−0.503	0.309	2.642	1	0.104	0.605	0.330	1.109
	Age	−0.438	0.315	1.931	1	0.165	0.645	0.348	1.197
	Literacy	0.414	0.252	2.689	1	0.101	1.513	0.922	2.481
Step 6	Gender	−0.550	0.307	3.203	1	0.074	0.577	0.316	1.054
	Literacy	0.518	0.240	4.639	1	0.031*	1.678	1.048	2.688

*p-value < 0.05.

## Discussion

Based on our knowledge, the current study is the first scientific report to examine the 5-year survival rate of patients with OSCC in southern Iran. The results of the current study indicate that age and occupation were associated with the overall 5-year survival rate in OSCC patients based on the log-rank test, while Cox regression analysis, after including all variables in the model and adjusting them, showed that all variables including age, gender, marital status, ethnicity, employment status, insurance status, and educational status were not associated with the overall 5-year survival rate. Further investigation provides potential explanations for these findings and discusses how these findings can be applied in clinical practice.

In this study, the 5-year survival rate in patients with OSCC was reported to be 44.84%. Gholizadeh et al. (8) reported a survival rate of 40.24%, Jafari et al. (10) reported a survival rate of 49.40%, and Tajmiririahi et al. (11) reported a survival rate 41.7% in OSCC patients. These studies have been conducted on the Iranian population in the last 5 years. The possible reason for the higher overall 5-year survival rate in the current study may be the potential advancements in diagnostic and therapeutic methods for OSCC in recent years. Additionally, the overall 5-year survival rate in the present study was lower compared to reports from some developed countries, e.g., the United States, but higher compared to survival rates in studies from developing countries, e.g., Uganda. For example, in the study by Zanoni et al. (12), the 5-year survival rate in 2,082 patients with OSCC was 64.3% in the United States, while in the study by Asio et al. (13) in Uganda, it was only 20.7%. The difference in the overall 5-year survival rate in this research compared to other studies in developed countries may be due to the better healthcare infrastructure and better access of patients to medical facilities in Iran.

In the current study, it has been shown that older patients with OSCC have a better overall 5-year survival rate compared to younger patients, but when other risk factors were adjusted, this association disappeared. Consistent with the current study, Oh et al. (14) and Chang et al. (15), who specifically investigated the impact of age on survival in patients with oral cancers, after examining data of 5,518 individuals found that age is not an independent predictor of overall survival in OSCC. Furthermore, in another study, which classified age relatively similar to the present study, the results also showed that age does not have a significant difference in the survival of patients with OSCC (16). However, there are contradictory findings, which report older patients’ worse survival due to comorbidities and adverse drug effects, while other studies have linked younger individuals to worse survival due to the CCND1 gene polymorphism (which is associated with the early onset of head and neck cancer) (17, 18). Overall, the impact of age on OSCC prognosis is still debatable (17). Although some studies have reached contradictory findings, they are not able to precisely explain the cause and pathological mechanism; therefore, further research with larger sample sizes is necessary.

The current study has shown that gender is not associated with 5-year survival rates in patients with OSCC. Similar to the current study, the studies by Siriwardena et al. (19), Lo et al. (20), Lee et al. (21), and Honorato et al. (22) did not find a significant relationship between survival rates and gender in patients with oral cancer. However, contradictory to this study, the study by Choi et al. (23) showed gender as an important factor in the prognosis and survival of OSCC cancer. Oh et al. (24) also introduced being female as the most important predictor of the overall 5-year survival rate in young and middle-aged non-smoking women. It seems that this disease is often diagnosed late, and at that time, background variables cannot play a significant role. However, it is suggested that for further investigations, cohort studies should be conducted to examine the role of gender through exploring behavioral, hormonal, genetic factors, and differences in immunological mechanisms between men and women.

The results of the current study indicated that employment type was associated with the overall 5-year survival rate in patients with OSCC in such a way that housewives had a higher survival rate compared to other occupational groups. However, in the multivariable analyses, this association was eliminated, suggesting the potential mediating factors in this relationship. Some studies have shown a positive impact of returning to work on the survival of head and neck cancer patients, which may be due to higher income, increased physical activity, and better emotional status, potentially having a positive effect on the survival of patients with oral cancer (15).

No association was found between education level and 5-year survival rate in patients with OSCC in the present study. The results of Asio’s study in Uganda (13) and in the Fengqiong Liu study in China on patients with oral cancer also showed no association between education level and survival rate (25). However, contrary to the current study, some scientists believe that oral cancer is strongly influenced by social factors (26, 27). For example, in Brazil and Taiwan, patients with a lower income and education level had a higher mortality rate due to oral cancer (26, 28). Furthermore, data from a study in India showed that patients with a lower educational background have lower survival rates for breast cancer and oral cavity cancers, which is attributed to having a more advanced stage of the disease at the time of diagnosis. On the other hand, education level may be considered as a substitute for socio-economic status, which can be related to survival (25). However, the current study did not find any association between education and the overall 5-year survival rate.

Marital status was not identified as an independent predictor of the overall 5-year survival rate in OSCC patients in the Cox regression analysis. Contrary to the current study, several studies have introduced marriage as a protective factor for the survival of patients (26, 29–32). These studies explained that married patients may receive support in adopting a healthy lifestyle and a positive perspective (30). Additionally, another study listed smoking as a factor for lower survival in unmarried individuals (29). Despite the above studies, the research team did not find a study consistent with the findings of the current study and suggests conducting further studies to examine the role of marital status in the overall 5-year survival rate of OSCC patients.

The current study has shown that insurance type is not related to survival rate. In contrast to the current study, other studies have shown the superiority of private insurance over Medicare and Medicare disability in the survival of patients with head and neck cancers (33). Another study has also shown that insurance status and household income level are important independent predictors of overall survival in throat squamous cell carcinoma (34). In other words, individuals with private insurance started early treatment and had much better outcomes compared to uninsured patients or recipients of Medicaid or Medicare. Perhaps the possible reason for not finding a correlation between the type of insurance and the overall 5-year survival rate in the present study is that this disease is often diagnosed late, and at that time, background variables cannot play a significant role as influential factors.

Finally, the current study showed that ethnicity is not a significant factor in relation to the overall 5-year survival rate of patients with OSCC. These findings were consistent with the findings of a study by Mahboubi et al. (35) on Asian and non-Asian cancer patients, which showed that Asian patients generally had worse outcomes compared to non-Asian patients. Additionally, another study showed that the survival rate of OSCC in black people was lower than that in white people. A comparison among Iranian ethnic groups also showed that Kurds had the lowest survival rate and Turks had the highest survival rate in colorectal cancer (36). The participants in the current study live in a province and have numerous interactions with each other, which may lead to similar lifestyles. Further studies are necessary to gain a comprehensive understanding of the complex interactions between ethnicity, environmental exposure, and genetic predisposition in OSCC with the overall 5-year survival rate. Studying diseases in different ethnic groups can help identify reasons.

This research was derived from the Khuzestan cancer registry system and published for the first time. On the other hand, this study had some limitations. To determine the vital condition, we called the phone numbers of the patients registered in the cancer registration system. Some phone numbers have been changed or incorrectly recorded. In addition, it was not possible to find all the patients due to immigration. The authors did not collect information regarding the treatments of the included patients and deleterious habits (such as smoking and alcohol use), tumor location, or TNM classification. Finally, the data of the cancer registration system do not include the clinical characteristics of patients such as tumor stage and pathological grade.

## Conclusion

Based on the findings of the present study, since none of the demographic variables were associated with the overall survival rate, efforts should continue to identify effective factors and investigate the relationship between pathological factors such as recurrence status, distant metastasis, type of treatment, and involved organs.

## Data Availability

The raw data supporting the conclusions of this article will be made available by the authors, without undue reservation.
